# Evaluation of reliability and validity of the Persian version of Peters et al. delusions inventory (PDI-40) in iranian non-clinical and clinical samples

**DOI:** 10.1186/s40359-023-01341-w

**Published:** 2023-09-27

**Authors:** Seyed Ruhollah Hosseini, Roghieh Nooripour, Nikzad Ghanbari, Abbas Firoozabadi, Emmanuelle Peters

**Affiliations:** 1 Department of Psychology, Faculty of Education Sciences and Psychology, Ferdowsi University of Mashhad, Mashhad, Iran; 2 Department of Counseling, Faculty of Education and Psychology, Alzahra University, Tehran, Iran; 3 Department of Clinical Psychology, Faculty of Psychology and Educational Sciences, Shahid Beheshti University, Tehran, Iran; 4 Department of Psychology, Institute of Psychiatry, Psychology & Neuroscience, King’s College London, London, UK

**Keywords:** Delusion, Psychotic-like experiences, Validity, Reliability

## Abstract

**Background:**

Some individuals may manifest psychotic symptoms that do not fulfill the requisite clinical criteria for a formal diagnosis of psychosis. The assessment of susceptibility to delusions, encompassing both clinical and non-clinical cohorts, frequently makes use of the Peters et al. Delusions Inventory (PDI-40). This study aimed to evaluate the reliability and validity of the Persian version of Peters et al. Delusions Inventory (PDI-40) in Iranian non-clinical and clinical samples.

**Methods:**

The present study employed a cross-sectional, correlational design in 2020. A total of 1402 Iranian participants were recruited for the study, which consisted of three distinct stages. The first stage involved an Exploratory Factor Analysis (EFA) conducted on a non-clinical sample of 512 participants. The second stage comprising different non-clinical sample 764 participants to perform a Confirmatory Factor Analysis (CFA). In the third stage, a clinical sample of 126 psychotic patients was compared to a non-clinical sample. All participants completed the PDI-40, the Community Assessment of Psychotic Experiences (CAPE-42), and the Depression, Anxiety, and Stress Scale (DASS-21). The internal structure of PDI-40 was examined through the analysis of its factor structure using LISREL 8.8.

**Results:**

The EFA analysis unveiled nine components within Persian version of PDI-40. The CFA analysis demonstrated an excellent fit of the nine-factor structure of Persian PDI-40 to the data. The total score exhibited high internal reliability, as indicated by Cronbach’s alpha coefficient of 0.92. Moreover, Persian PDI-40 exhibited satisfactory evidence of convergent validity, as significant correlations were observed between dimensions of PDI-40 and subscales of CAPE-42 and DASS-21. Lastly, findings indicated that psychotic participants scored higher than non-clinical participants in all components of the PDI-40(p < 0.05).

**Conclusion:**

Persian version of the PDI-40 demonstrates strong reliability and validity for assessing delusion proneness in both non-clinical and clinical samples in Iran. The observed distinctions between psychotic and non-clinical participants underscore its potential as a valuable tool for discerning delusion proneness in diverse contexts.

## Introduction

According to the American Psychiatric Association [1] delusions are classified as a critical criterion for identifying psychotic disorders. The DSM-5 defines delusions as persistent, false beliefs, which endure despite contradictory evidence or rational discourse. They are prominent sign of several psychiatric ailments, for example, schizophrenia, schizoaffective disorder, or delusional disorder. These convictions are neither customary nor cultural or religious in origin and cannot be justified by an individual’s personal or cultural background or religious beliefs. Delusions can be bizarre or non-bizarre in nature. Bizarre delusions are implausible and not understandable within the individual’s cultural context, while non-bizarre delusions involve situations that could potentially occur in real life, though they are false [2].

While delusions are commonly recognized as a defining characteristic of psychosis according to contemporary diagnostic frameworks for mental disorders, numerous recent investigations have revealed that significant segments of non-clinical individuals may encounter these symptoms at various stages throughout their lifetimes [3–5].

A recent study conducted to evaluate the prevalence of anomalous perceptual experiences within a substantial cohort of individuals from the non-clinical population revealed that individuals without a history of hallucinations, yet possessing delusional beliefs, exhibited no significant distinctions in comparison to non-clinical counterparts across various indices measuring such experiences [6–8]. A study demonstrated that the occurrence of delusions does not depend on anomalous perceptual experiences [9]. They demonstrated that the occurrence of delusions is not contingent upon anomalous perceptual experiences. The findings revealed that, while the worry induction procedure led to a notable increase in levels of worry, it did not influence working memory or the tendency to jump to conclusions. However, the induction of worry did result in heightened mild anomalous experiences, including sensations of unreality, perceptual changes, and temporal disintegration [9]. Interestingly, the study did not find a significant effect of worry on the occurrence of hallucinations. Therefore, the study indicates that a period of heightened worry can lead to subtle perceptual disturbances, which have been identified as factors contributing to an increased likelihood of delusions. A notable proportion of individuals who are not clinically diagnosed exhibit symptoms commonly associated with delusional disorders [10]. This finding challenges the conventional belief that delusions exclusively manifest in clinical populations characterized by severe mental health disorders. One potential explanation for this phenomenon is that delusional symptoms exist on a continuum, with varying degrees of severity and pervasiveness across the non-clinical population [11, 12].

Given the hypothesis of subclinical manifestations of delusions [13], a significant proportion of the non-clinical population may experience varying degrees of Psychotic-Like Experiences (PLEs) that do not qualify as symptoms of a psychotic disorder [14–16]. Therefore, studying the nature and prevalence of these non-clinical population experiences has led to more research attention during recent decades.

The term “delusion” is employed to characterize a range of PLEs or subclinical psychotic symptoms that arise in the absence of overtly clinically significant psychiatric disorders [17]. PLEs encompass a spectrum of phenomena, including hallucinations and delusions, which manifest without meeting the diagnostic criteria for a full-blown psychotic disorder. It is important to note that while delusions are a component of PLEs, PLEs encompass a broader array of experiences beyond just delusions [18]. These PLEs have been identified as significant in various studies [19, 20], underscoring the importance of understanding their nature and potential implications [21]. These instances, integral constituents of the extensive realm of psychosis, proffer the notion that individuals within the non-clinical stratum can potentially confront such manifestations bereft of consequential distress or impairment that would substantiate a clinical categorization. The elucidation of this phenomenon contributes to a nuanced understanding of the intricate interplay between psychopathological attributes and their varying degrees of clinical gravity [22].

PLEs, often characterized by perceptual distortions, delusional ideation, and disorganized thinking, have garnered considerable attention due to their potential relevance to clinical psychosis [23]. Prior studies have consistently unveiled intriguing associations between PLEs and emotional symptoms, suggesting a bidirectional relationship wherein emotional distress might potentiate the emergence of certain perceptual anomalies [24, 25]. Conversely, anomalous experiences may contribute to heightened emotional turbulence, including symptoms of stress, depression, and anxiety [26, 27]. This intricate interdependence underscores the necessity for a comprehensive investigation into the shared etiological underpinnings, neurobiological mechanisms, and potential therapeutic interventions that could address both facets holistically. Through a concerted effort to synthesize empirical findings from both domains, this study aims to contribute to a deeper comprehension of the dynamic interrelationship between PLEs and emotional symptoms, enriching our insights into the broader landscape of psychological well-being and psychopathology [24]. Recent research has concentrated its efforts on investigating subclinical psychotic experiences, with the primary objective of elucidating the intricate mechanisms that contribute to the escalation of these subtle symptoms and their consequent influence on the broader spectrum of mental well-being [28, 29]. Some studies such as McGrath et al., [30], the meta-analysis conducted by Van et al., [12], and the meta-analysis conducted by Linscott et al., [31] have illuminated a prevalence of 5.8%, 3.1%, and 7.2% respectively, for PLEs in non-clinical populations. A recent study by Bourgin et al., [32] the lifetime prevalence of PLEs demonstrated a noteworthy elevation among women [33], younger individuals, the unemployed, the non-married, those with higher educational attainment, and those from lower-income families. Research has indicated instances of such experiences within normal populations as well, shedding light on the spectrum of human perceptual and cognitive variations [34, 35]. By examining these phenomena within a non-clinical context, we gain valuable insights into the potential early manifestations and developmental trajectories of psychosis-related symptoms. Furthermore, exploring the presence of these experiences in young individuals from the general population holds significant implications for understanding the origins and risk factors associated with psychosis. Therefore, a comprehensive examination of the continuum of psychosis should encompass both non-clinical and clinical samples, allowing for a more nuanced understanding of these phenomena and their relevance in diverse settings. Moreover, another study showed the significant distress and impairment associated with PLEs [30, 36].

Heilskov et al., [3] proposed the psychosis continuum hypothesis, which suggests that when an adequate number of symptoms reach a specific threshold, it culminates in the emergence of psychosis. Consequently, the perception of delusions manifests variations along dimensions encompassing conviction, preoccupation, distress, and functional impact. The operationalization of these dimensions is advisable to discern benign, atypical, and clinically substantial beliefs, departing from a binary perspective of veracious or fallacious convictions. The existence of diverse definitions, evaluation instruments, and conceptual frameworks pertaining to PLEs has yielded inconsistent outcomes in relation to their prevalence, persistence rates, and clinical ramifications.

One of the instruments developed to measure psychotic symptoms in the non-clinical population is the Peters et al. Delusions Inventory (PDI: Peters, Joseph et al., [37]. It has been developed to measure psychotic symptoms in non-clinical population. Initially designed to evaluate delusional thinking in non-clinical population and assess sensitivity of non-clinical samples to various delusions, the PDI-40 consists of 40 items. It assesses multiple dimensions, including illusions of control, misinterpretations, misidentifications, reference, persecution, expansiveness, being influenced, guilt, depersonalization, and hypochondriasis [37]. The PDI-40 serves as a widely used tool for measuring delusion proneness in non-clinical population, utilizing present state examination as a template. It incorporates dimensions that evaluate distress, preoccupation, and conviction of unusual beliefs. Apart from determining the presence or absence of each belief, the PDI-40 also measures the level of distress caused by the belief, the degree of mental focus on the belief, and the individual’s confidence in these beliefs [38]. The PDI-40 has found common usage in assessing the likelihood of subclinical psychosis in specific populations, such as twins [39], members of a particular religion [40], cannabis users [41], and parents of patients with schizophrenia and bipolar diagnoses [42, 43]. In addition to its widespread usage, the PDI-40 has been translated into multiple languages and its psychometric properties have been studied. Translations of the PDI-40 have been conducted in various languages, including Italian [44], Polish [45], Spanish [46], Japanese [47], Korean [48], and Taiwanese [49]. These translations have enabled researchers from different linguistic backgrounds to utilize the PDI-40 in their studies and examine its reliability and validity in diverse cultural contexts.

At present, the existing body of scholarly inquiry pertaining to the distinct attributes of the PDI-40 remains delimited, with a distinct emphasis placed upon the elucidation of sensitivity and specificity thresholds. It is noteworthy that a solitary investigation has undertaken an exploration of the differentiation between cohorts comprising non-clinical and clinical samples. The outcomes of this endeavor have revealed a sensitivity rate of 74%, concurrently accompanied by a specificity rate of 79% [50].

While the PDI-40 has undergone translation and scrutiny in numerous languages, a conspicuous research gap persists regarding its application in the Persian language. To address this pronounced void, our study is resolutely dedicated to accomplishing the translation of the PDI-40 into Persian, followed by subjecting it to an intricate process of cross-validation. This meticulous undertaking will encompass the utilization of both non-clinical and clinical cohorts originating from Iran. Our investigation is the establishment of a robust and culturally sensitive rendition of the PDI-40 in the Persian language, accompanied by a comprehensive assessment of its psychometric properties within the specific context of Iran. By embarking upon this pivotal research initiative, our intent is unequivocally focused on contributing invaluable insights to the field of cross-cultural investigations concerning delusion proneness. Simultaneously, our efforts aim to significantly enrich the evaluative framework for psychotic symptoms within the Iranian population.

In light of the above, this study aimed to evaluate the reliability and validity of the Persian version of Peters et al. Delusions Inventory (PDI-40) in Iranian non-clinical and clinical samples. By assessing the psychometric properties of this inventory, we will provide a comprehensive understanding of susceptibility to various forms of delusions in Iranian cultural context.

## Materials and methods

We used a cross-sectional, correlational study in 2020. A total of 1402 Iranian participants were recruited for this study, which comprised three distinct stages. The first stage involved a non-clinical sample of n = 512 individuals, with a mean (SD) age of 30.17 (11.35), and aimed to conduct Exploratory Factor Analysis (EFA) on the Persian version of the PDI-40. In the second stage, a different non-clinical sample of n = 764 individuals, with a mean (SD) age of 28.26 (12.73), was recruited for Confirmatory Factor Analysis (CFA). Participants in these both stages completed the Persian version of the PDI-40, as well as additional measures such as the Community Assessment of Psychotic Experiences (CAPE-42) and the Depression, Anxiety, and Stress Scale (DASS-21). Lastly, a clinical sample of n = 126 psychotic patients, with a mean (SD) age of 24.02 (3.63), was included in the study to compare the PDI scores between non-clinical and clinical samples. In this stage, the clinical sample completed Persian version of the PDI-40 for comparing the PDI scores between non-clinical and clinical samples.

In the realm of the non-clinical sample, the initiative of participant recruitment was underpinned by a comprehensive embrace of diverse online platforms situated within the landscape of Iran. This multifaceted array encompassed an array of channels, including, yet not confined to, Internet advertisements, email outreach, Instagram, Telegram, WhatsApp, an assortment of forums, social networks, and programs designed for the facilitation of short message services (SMS). It is noteworthy to mention that this intricate recruitment strategy was bolstered through a concomitant methodological facet, wherein in-person questionnaires were adroitly harnessed as adjunctive avenue for participant engagement.

Turning our focus to the domain of clinical sample recruitment, the process was characterized by the meticulous identification and selective inclusion of individuals endowed with well-established psychiatric diagnoses. This discerning procedure unfolded within the precincts of a hospital setting, wherein a discerning stratagem was applied. To elaborate, a purposive selection approach was deployed, targeting patients who exhibited an array of psychiatric symptoms, encompassing diverse manifestations such as delirium, hallucinations, disordered or catatonic behaviors, disorganized speech, aberrant emotional states, and discernible functional decline. This scrupulous procedure culminated in the judicious inclusion of a cohort of 126 patients, effectively enrolling them into the study’s framework. This methodological rigor ensured the alignment of participant clinical profiles with the predefined research objectives, thus endowing the subsequent analytical pursuits with elevated validity and contextual relevance.

### Procedure

The process of translating the PDI-40 into Persian, while maintaining its conceptual accuracy, was meticulously conducted using a widely acknowledged technique known as translation and back-translation. This method ensures the fidelity of the translated instrument by involving two distinct and independent translation teams: one responsible for translating the measure into the target language, and the other for proficiently translating it back into the original language.

To achieve this, our study engaged a panel of three proficient translators, each working independently to minimize potential variations in their interpretation and presentation of the scale’s constituent items. This approach was carefully chosen to ensure the robustness of the translation process. We took deliberate measures to optimize the clarity and comprehensibility of the translated version. To achieve this, a distinguished scholar specializing in English studies meticulously reviewed and refined the translation as required.

In line with the highest standards of cross-cultural adaptation, we took into account the guidelines and technical recommendations for adapting tests to multiple languages and cultures, as outlined by authoritative sources such as the International Test Commission (ITC) Guidelines for Translating and Adapting Tests. This comprehensive approach helped us to align the translated version of the PDI-40 with its original intent, while catering to the linguistic and cultural nuances of the Persian-speaking population.

We ensured that the translated items’ lengths were carefully balanced to mirror those of the original scale, preserving the inherent meaning and intended purpose of each individual item. This attention to detail was essential to maintaining the scale’s psychometric properties and the validity of the data collected through its application in the Persian-speaking context.

Our translation and back-translation process adhered to established best practices and methodological rigor, combining linguistic expertise, scholarly review, and cross-cultural considerations to create a precise and conceptually congruent Persian version of the PDI-40.

It is essential to highlight that the survey in question, a pivotal component of this endeavor, was conducted in the year 2020. Collectively, the meticulously detailed and well-structured translation and adaptation process outlined herein reflects a methodical and rigorous approach, culminating in the achievement of a translated PDI-40 scale in Persian that consistently upholds its intended validity and efficacy.

Participants were required to meet specific inclusion criteria to be eligible for the study. Firstly, they needed to demonstrate proficiency in reading and writing Persian (Farsi), the primary language of the study. Additionally, completion of high school was necessary to ensure a certain level of education among participants. Furthermore, participants needed to be residents of Iran to align with the geographical focus of the research. Lastly, fluency in Farsi was another criterion to ensure effective communication and understanding throughout the study. The only inclusion criterion for the clinical sample was experiencing the first onset of any symptoms of psychosis. These inclusion criteria were deliberately broad as the study aimed to explore various aspects of the research topic.

In alignment with our dedication to transparency, ethical standards, and the protection of participants’ rights, we meticulously ensured the acquisition of written informed consent from all parties involved. Prior to their participation in the survey, explicit written informed consent was obtained from all participants, except those with psychosis. For psychotic patients, their families or legal guardians assumed the responsibility of providing consent on their behalf. We acknowledge the need for additional elaboration on two key aspects highlighted by the reviewer. First and foremost, the evaluation of our study by an accredited Ethics Committee or Research Board constitutes an integral facet of our ethical framework. We apologize for the oversight in not explicitly delineating this process in our initial procedural description. It is imperative to emphasize that our study’s design and consent procedures underwent rigorous review and garnered approval from the relevant Ethics Committee or Research Board. This oversight has been rectified by explicitly specifying the involvement of these authoritative bodies in the ethical evaluation of our research. Secondly, we recognize the necessity of providing a more comprehensive justification for our decision to obtain informed consent from the parents or legal guardians of participants with psychosis, rather than seeking direct consent from the participants themselves. This decision was made in accordance with the stipulations set forth by the Ethics Committee or mandated by local legislation, which prescribe that participants with compromised decision-making capacity necessitate a surrogate decision-maker to grant consent on their behalf. By aligning our consent procedures with established guidelines, we aimed to ensure the ethical treatment and safeguarding of vulnerable participants. It is essential to clarify that while consent was procured from parents or legal guardians, participants themselves were afforded the opportunity to express verbal assent, and this assent procedure was meticulously documented. This approach strikes a judicious balance between ethical considerations and the acknowledgment of participants’ autonomy within the confines of their capacity.

### Measures

The **Peters Delusions Inventory-40 (PDI-40)** was selected as the primary measure to comprehensively assess delusional ideation in both non-clinical and clinical samples, over the abbreviated 40-item PDI (PDI-40) [40]. The PDI-40 [51] is a self-reported questionnaire consisting of 40 items designed to evaluate three key dimensions of delusional thinking: distress, preoccupation, and conviction. Participants indicate their level of distress, frequency of thoughts, and degree of belief for each endorsed delusional idea using a five-point scale. The inventory’s items are categorized into eight groups based on delusions identified in the Present State Examination [40]. The PDI-40 generates four distinct scores: total, distress, preoccupation, and conviction. The total score is derived by assigning a value of 1 to each affirmative response and 0 to each negative response, yielding a maximum possible score of 40. Additionally, when an item receives an affirmative response, participants rate each dimension on a scale of one to five. Previous research has demonstrated favorable reliability (α = 0.88, test-retest reliability = 0.82) and validity of the PDI-40 [38]. In the context of the current study, the calculated Cronbach’s alpha coefficient for the PDI-40 instrument is 0.85. This statistical value denotes the internal consistency reliability of the PDI-40’s items in this study.

***Community Assessment of Psychotic Experiences (CAPE-42)***: The Community Assessment of Psychotic Experiences (CAPE-42) is a self-report questionnaire adapted from Peters et al.‘s Inventory of Deceptions (2004) [51]. It was specifically developed to gauge the prevalence of psychotic experiences within the non-clinical population over their lifetime [52]. Extensive research has substantiated the reliability and validity of self-reported psychotic experiences as captured by this questionnaire [53]. Comprising 42 items, the CAPE questionnaire encompasses three distinct symptom dimensions: positive (20 items), depressive (eight items), and negative (14 items). Owing to inadequate reliability of self-report measures, symptoms related to mania and disorganization were excluded from the questionnaire [54]. For measuring psychotic-like symptoms, the CAPE employs two 4-point Likert scales to assess both the frequency and associated distress of each symptom. Studies have indicated that the three dimensions measured by the CAPE are independent yet correlated, demonstrating satisfactory internal consistencies [55], and adequate divergent validity [53]. In Iran, specific studies have reported Cronbach’s alpha values of 0.93 for the total score, and 0.91, 0.71, and 0.88 for the positive, negative, and depressive factors, respectively [56]. In the current study, internal consistency reliability, as assessed by Cronbach’s alpha coefficients, yielded of 0.73 for the total score, and 0.81, 0.78, and 0.73 for the positive, negative, and depressive factors, respectively.

#### The Depression, anxiety, and stress scale − 21 items (DASS-21)

Lovibond and Lovibond developed DASS-21 in 1995 as a measure of anxiety, depression, and stress. This scale consists of 21 items, with each component represented by seven items. The scoring system for each item ranges from zero (indicating; “it does not apply to me” or “never”) to three (indicating; “applies to me very often”) [57]. Therefore, the possible range for each sub-scale is from zero to 21. Brown et al. [58] reported a validity coefficient of 0.77 for the DASS-21, indicating its satisfactory validity. In Iran, the scale has demonstrated a validity coefficient of 0.82, as determined by Cronbach’s alpha [59].

### Statistical analysis

This research encompassed three distinct stages of analysis. In the initial stage, an Exploratory Factor Analysis (EFA) was conducted using Principal Component Analysis (PCA) with varimax rotation. The indicators used to evaluate model fit during this stage included the Kaiser-Meyer-Olkin (KMO) measure of sampling adequacy and the Bartlett’s test of sphericity. The suitability of the data for factor analysis was determined by these indicators, with a KMO value above 0.7 and a significant Bartlett’s test suggesting a valid factor analysis. The Eigenvalues and scree plot were examined to identify the number of factors to retain, based on the inflection point in the scree plot. In the second stage, Confirmatory Factor Analysis (CFA) was performed to validate and confirm the factor structure of the questionnaire. The fit of the CFA model was assessed using various indicators, including the Comparative Fit Index (CFI), the Root Mean Square Error of Approximation (RMSEA), and the Standardized Root Mean Square Residual (SRMR). To establish model fit, the CFI values should ideally exceed 0.90, while the RMSEA and SRMR values should be below 0.08. These benchmark values are widely accepted in the literature [60]. The reliability of the questionnaire was assessed using the test-retest procedure, which involves administering the questionnaire to the same participants at two different time points to assess the stability of scores over time. The normality of the variables was tested using the Kolmogorov-Smirnov test, with a significance level set at p < 0.05. This test assesses whether the data follows a normal distribution, which is an assumption of many statistical analyses. Demographic characteristics and the Pearson correlation between the Persian versions of PDI-40, DASS-21, and Psychotic Experiences-42 (CAPE-42) were analyzed using SPSS Version 22.0. Convergent and divergent validity were assessed by examining the correlations between the PDI-40 scores and scores on the other measures. Convergent validity is supported when the PDI-40 scores show significant positive correlations with related constructs, such as depressive and anxious symptoms, as measured by the DASS-21. Conversely, divergent validity is supported when the PDI-40 scores have weaker or non-significant correlations with constructs that are theoretically distinct, such as psychotic experiences as measured by the CAPE-42. In the third stage of the study, a comparison was made between the PDI-40 scores of non-clinical and clinical samples. This was achieved through appropriate statistical tests, such as t-tests or Mann-Whitney U tests, depending on the distribution of the data and the assumptions being met. The internal structure of the PDI-40 was further assessed through the analysis of its factor structure using AMOS-22. Model fit indices similar to those used in the CFA stage were examined to ensure the adequacy of the factor structure. Receiver Operating Characteristic (ROC) curve analysis was conducted to assess the diagnostic accuracy of the PDI-40 in discriminating between non-clinical and clinical samples.

## Findings

**Descriptive statistics**.

A total of 126 Psychoses and 1268 non-clinical Iranian individuals participated in the research (Table 1). The mean age was 28.40 (SD = 10.26). In terms of gender distribution in non-clinical sample, 829 (65.37%) participants were female, and 439 (34.62%) were male. Regarding marital status, 751 (59.22%) participants reported being single, 482 (38.01%) reported being married, and 35 (2.76%) did not provide a response to this question.

**Table 1:** Descriptive statistical data for PDI-40 base gender (male/female) in the Psychosis and non-clinical sample

	Non-clinical (n = 1276)								Psychosis (n = 126)								
	Female (n = 829)				Male (n = 439)				Female (n = 68)				Male (n = 58)				
	M	SD	K	SK	M	SD	K	SK	M	SD	K	SK	M	SD	K	SK	
**F1**	31.75	25.40	-0.41	0.73	29.32	23.01	-0.21	0.66	50.11	27.73	-0.23	0.32	60.86	30.39	-0.76	-0.06	
**F2**	10.49	12.78	0.54	1.21	9.29	11.85	2.43	1.55	36.55	21.47	0.62	0.98	41.72	21.61	0.05	0.45	
**F3**	7.22	9.70	1.55	1.44	5.34	8.29	2.64	1.76	15.05	12.14	0.03	0.52	18.86	14.97	-0.89	0.52	
**F4**	11.44	13.66	0.99	1.25	9.11	12.87	3.16	1.80	10.44	10.92	0.09	0.90	9.17	11.77	0.79	1.21	
**F5**	9.80	12.95	2.28	1.62	9.92	13.68	3.70	1.88	17.50	12.74	-0.71	0.24	17.82	16.77	-0.19	0.84	
**F6**	4.05	6.89	4.32	2.00	4.61	7.43	5.68	2.16	14.58	12.76	-1.10	0.35	22.65	17.86	-0.59	0.66	
**F7**	4.74	6.42	1.63	1.39	3.55	5.42	2.39	1.60	6.00	8.325	4.67	1.89	12.24	11.91	-0.78	0.52	
**F8**	2.75	5.12	3.34	1.94	3.57	6.10	4.18	1.96	6.14	5.869	-0.87	0.35	6.31	7.21	1.11	1.15	
**F9**	120.15	90.74	0.02	0.78	113.56	89.31	0.75	0.96	5.08	6.163	0.17	0.93	9.06	8.08	-1.06	0.31	
**PDI**	37.86	28.91	-0.83	0.44	38.81	30.06	-0.63	0.60	161.50	84.26	1.11	0.73	198.72	108.32	0.14	0.68	

*Note.* PDI = Peters et al. Delusions Inventory; M = Mean, SD = Standard Deviation, K = Kurtosis, SK = Skewness, F1-9 = components of PDI; F1 = grandiosity; F2 = persecution; F3 = control; F4 = depersonalization; F5 = catastrophic ideation and thought broadcast; F6 = negative self; F7 = suspiciousness; F8 = thought disturbance; F9 = ideation of reference

### Factor structure

### Item analysis

To assess internal consistency, inter-item and item-total correlations were computed for the PDI-40. Out of the 809 inter-item correlations examined, 805 were found to be statistically significant. All item-total correlations were found to be statistically significant, with values ranging from 0.33 to 0.69. It is crucial to emphasize that none of the items displayed a negative or remarkably low correlation with the total score. Thus, based on the comprehensive item analysis, there was no necessity to exclude any items from the questionnaire.

### Exploratory factor analysis (EFA)

To investigate the internal structure of the PDI-40 within a non-clinical Iranian population consisting of 512 individuals, an Exploratory Factor Analysis (EFA) was performed. Principal Component Analysis (PCA) with varimax rotation was employed for this analysis. Moreover, the PCA demonstrated that all factors loaded onto the nine components exceeded 0.50, with the exception of items 18, 23, 26, and 32 (Table 2).

**Fig. 1:**
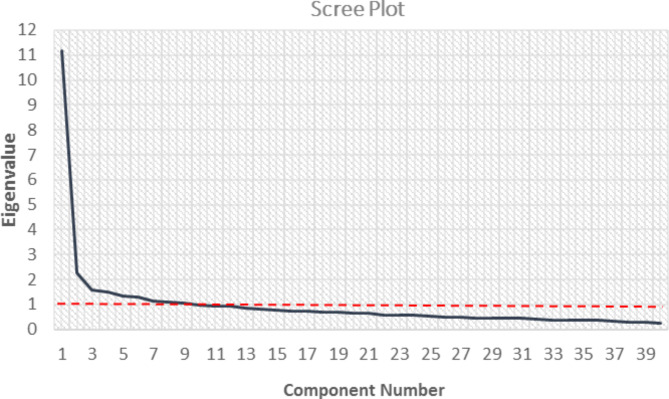
Scree plot of eigenvalues from the parallel and principal component analysis

The results of the EFA revealed a Kaiser-Meyer-Olkin (KMO) index of 0.92, surpassing the recommended minimum threshold of 0.6. This index value indicates a high level of sampling adequacy, affirming the suitability of the sample size for conducting factor analysis in the specified population. Therefore, these findings provide robust support for the appropriateness of the collected data to carry out factor analysis in this particular population [61]. Inter-item correlations calculated the results showed that except for the all correlation between items were statistically significant except relationship between item7 with item 26, 7 with 27 and 33 with 25. Additionally, a significant Bartlett’s Test of Sphericity [62] was observed (χ2 = 7332.9, p < 0.001), indicating that the correlation matrix was suitable for factor analysis. Examining the scree plot revealed a distinct break after the nine components (Fig. 1). This finding was further supported by additional analyses, which indicated that the Eigenvalues for these nine components exceeded the corresponding criterion values derived from a randomly generated data matrix of the same size (40 variables × 512 respondents). Collectively, these nine components accounted for 56.03% of the total variance (Table 2).

**Table 2:** EFA of 40-items PDI-40 (n = 512)

Items			Factor Loading	Cronbach’s Alpha if Item Deleted	Corrected Item-Total Correlation	Eigenvalue		
	M	SD				Total	% of Variance	Cumulative %
1	2.71	4.20	0.643	0.930	0.523	11.156	27.890	27.890
2	0.85	2.75	0.514	0.931	0.391	2.263	5.658	33.548
3	0.92	2.89	0.645	0.931	0.395	1.588	3.970	37.517
4	2.26	4.18	0.604	0.931	0.475	1.491	3.727	41.244
5	2.88	4.62	0.504	0.930	0.564	1.345	3.362	44.606
6	5.09	5.05	0.605	0.929	0.632	1.278	3.195	47.801
7	0.66	2.24	0.595	0.932	0.311	1.129	2.822	50.624
8	3.66	4.81	0.547	0.930	0.566	1.108	2.769	53.393
9	7.80	5.04	0.518	0.930	0.582	1.055	2.637	56.030
10	1.03	2.92	0.503	0.931	0.396	0.993	2.483	58.513
11	3.37	4.88	0.684	0.929	0.616	0.939	2.348	60.861
12	4.03	5.02	0.691	0.929	0.656	0.927	2.317	63.178
13	2.43	4.44	0.595	0.930	0.555	0.856	2.140	65.318
14	0.62	2.37	0.635	0.932	0.299	0.824	2.061	67.379
15	2.70	4.46	0.519	0.930	0.593	0.790	1.975	69.354
16	4.06	4.99	0.661	0.930	0.581	0.740	1.850	71.204
17	4.30	4.60	0.534	0.930	0.545	0.726	1.816	73.020
18	3.12	4.46	0.479	0.931	0.488	0.700	1.750	74.770
19	4.48	4.78	0.509	0.931	0.452	0.695	1.738	76.508
20	1.73	3.66	0.529	0.931	0.444	0.649	1.622	78.130
21	4.59	4.87	0.543	0.931	0.457	0.636	1.589	79.719
22	4.55	4.38	0.575	0.930	0.565	0.589	1.472	81.190
23	3.78	4.53	0.405	0.931	0.453	0.578	1.446	82.636
24	2.47	4.07	0.542	0.930	0.600	0.567	1.417	84.053
25	3.37	4.56	0.598	0.931	0.488	0.544	1.361	85.414
26	3.18	4.50	0.492	0.931	0.489	0.508	1.269	86.683
27	2.90	4.96	0.639	0.932	0.394	0.498	1.244	87.928
28	0.92	3.04	0.567	0.932	0.353	0.455	1.137	89.065
29	1.50	3.67	0.553	0.931	0.409	0.451	1.128	90.193
30	2.03	3.98	0.537	0.931	0.429	0.444	1.109	91.302
31	1.59	3.94	0.560	0.931	0.409	0.432	1.080	92.383
32	2.41	4.28	0.402	0.930	0.525	0.404	1.011	93.394
33	2.06	4.08	0.556	0.932	0.366	0.386	0.965	94.359
34	0.78	2.74	0.625	0.932	0.352	0.380	0.950	95.309
35	2.30	4.14	0.461	0.931	0.397	0.359	0.899	96.207
36	1.65	3.77	0.600	0.931	0.417	0.351	0.877	97.085
37	1.72	3.78	0.599	0.930	0.522	0.327	0.818	97.903
38	2.57	4.23	0.514	0.930	0.590	0.309	0.773	98.677
39	1.81	3.82	0.517	0.930	0.516	0.282	0.704	99.380
40	2.34	4.02	0.614	0.930	0.596	0.248	0.620	100.00

### Confirmatory factor analysis (CFA)

A total of 764 non-clinical individuals participated in Study 2. The mean age was 28.26 (SD = 12.73). In terms of gender, 489 participants (64.0%) identified as female, while 275 participants (36.0%) identified as male. Regarding marital status, 486 participants (63.61%) reported being single and 278 participants (36.38%) reported being married.

**Fig. 2:**
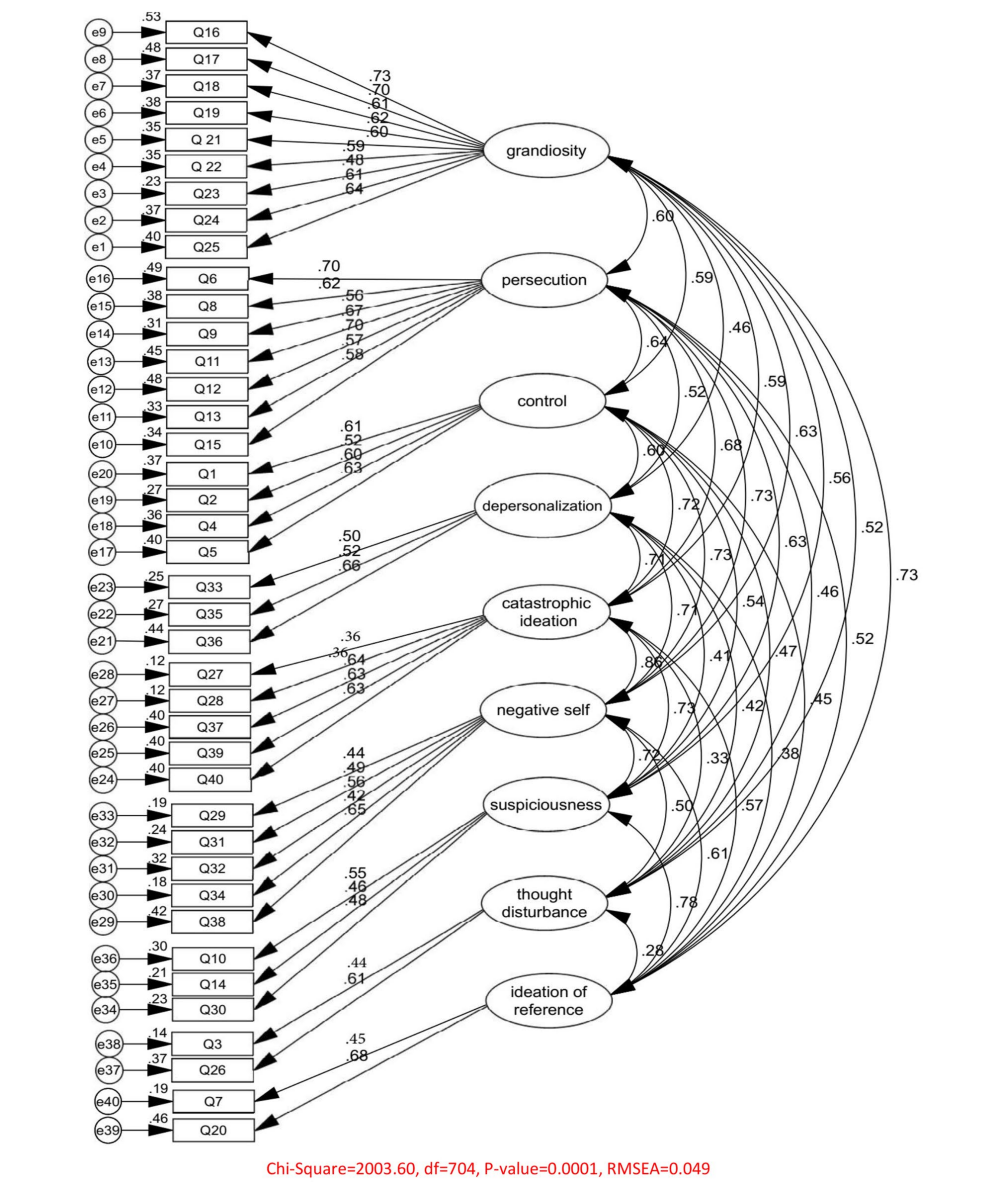
Model Fit indexes. Note.  = grandiosity; 2 = persecution; 3 = control; 4 = depersonalization; 5 = catastrophic ideation and thought broadcast; 6 = negative self; 7 = suspiciousness; 8 = thought disturbance; 9 = ideation of reference.

Confirmatory Factor Analysis (CFA) was conducted, employing maximum likelihood estimation, to evaluate the goodness of fit of the nine-factor solution derived from the Exploratory Factor Analysis (EFA) of PDI-40 [63]. The CFA was conducted on a sample of 764 non-clinical individuals. The initial CFA model included the nine factors and the 40-item solution derived from EFA. The final CFA model confirmed presence of nine factors (see Table 3; Fig. 2).

**Table 3:** CFA and Standardized Regression of 40-item PDI-40 (n = 764)

items			SRW	FL	T-value	P
q25	<---	grandiosity	0.636	0.64	19.24	***
q24	<---	grandiosity	0.609	0.61	18.28	***
q23	<---	grandiosity	0.477	0.48	13.70	***
q22	<---	grandiosity	0.591	0.59	17.56	***
q21	<---	grandiosity	0.596	0.60	17.77	***
q19	<---	grandiosity	0.615	0.62	18.52	***
q18	<---	grandiosity	0.606	0.61	18.18	***
q17	<---	grandiosity	0.695	0.70	21.60	***
q16	<---	grandiosity	0.725	0.73	22.80	***
q15	<---	persecution	0.583	0.58	16.74	***
q13	<---	persecution	0.574	0.57	16.46	***
q12	<---	persecution	0.695	0.70	21.59	***
q11	<---	persecution	0.671	0.67	20.47	***
q9	<---	persecution	0.560	0.56	16.43	***
q8	<---	persecution	0.615	0.62	18.60	***
q6	<---	persecution	0.701	0.70	21.57	***
q5	<---	control	0.631	0.63	17.64	***
q4	<---	control	0.597	0.60	16.53	***
q2	<---	control	0.521	0.52	14.19	***
q1	<---	control	0.608	0.61	16.83	***
q36	<---	depersonalization	0.663	0.66	16.69	***
q35	<---	depersonalization	0.518	0.52	13.11	***
q33	<---	depersonalization	0.500	0.50	12.60	***
q40	<---	catastrophic	0.632	0.63	18.18	***
q39	<---	catastrophic ideation and thought broadcast	0.634	0.63	18.29	***
q37	<---	catastrophic ideation and thought broadcast	0.635	0.64	18.35	***
q28	<---	catastrophic ideation and thought broadcast	0.346	0.36	9.30	***
q27	<---	catastrophic ideation and thought broadcast	0.351	0.36	9.45	***
q38	<---	negative self	0.648	0.65	18.97	***
q34	<---	negative self	0.421	0.42	11.75	***
q32	<---	negative self	0.563	0.56	16.14	***
q31	<---	negative self	0.488	0.49	13.78	***
q29	<---	negative self	0.441	0.44	12.44	***
q30	<---	suspiciousness	0.477	0.55	12.27	***
q14	<---	suspiciousness	0.458	0.46	10.78	***
q10	<---	suspiciousness	0.546	0.55	13.68	***
q26	<---	thought disturbance	0.612	0.61	10.65	***
q3	<---	thought disturbance	0.378	0.44	9.68	***
q20	<---	ideation of reference	0.679	0.68	14.69	***
q7	<---	ideation of reference	0.436	0.45	10.87	***

*Note.* RSW = Standardized Regression Weights, FL = Factor Loading, ***=P < 0.001,

**Table 4:** Confirmatory Factor Analysis (CFA) and Fit indexes for a nine-factor structure (n = 764)

model		RMSEA (CI 90%)	X^2^	SRMR	CFI	NFI	IFI	RFI	AGFI	GFI
non-clinical sample	Nine factors model	0.049 (0.047–0.052)	2003.6	0.049	0.97	0.92	0.97	0.95	0.86	0.89
female	Nine factors model	0.050 (0.047–0.053)	1565.51	0.054	0.96	0.93	0.96	0.93	0.84	0.86
male	Nine factors model	0.066(0.061–0.070)	1528.78	0.064	0.95	0.91	0.95	0.90	0.74	0.78
non-clinical sample	Single factor	0.078(0.075–0.080)	4144.94	0.060	0.94	0.92	0.94	0.92	0.76	0.79

***Legend***: X^2^ = Chi-Square, RMSEA: Root Mean Square Error of Approximation; SRMR: Standardized RMR; CFI: Comparative Fit Index; NFI: Normed Fit Index; IFI: Incremental Fit Index; RFI: Relative Fit Index; AGFI: Adjusted Goodness of Fit Index; GFI: Goodness of Fit Index.

The CFA results for the single factor, nine-factor structure and factor structure in male and female are presented in Table 4. The obtained results showed a better fit of the nine-factor model comparison single-factor model. Overall, the findings indicate good model fit, as the factor loadings for most items exceeded 0.40. However, items 27 and 28 had a factor loading of 0.36, slightly below the threshold. Model fit was evaluated using several fit indices, including the Root Mean Square Error of Approximation (RMSEA), Standardized Root Mean Square Residual (SRMR), Comparative Fit Index (CFI), Normed Fit Index (NFI), Incremental Fit Index (IFI), Relative Fit Index (RFI), Adjusted Goodness of Fit Index (AGFI), and Goodness of Fit Index (GFI) [64]. The criteria for these fit indices are as follows: RMSEA < 0.08 (with a 90% confidence level), SRMR < 0.09, CFI > 0.90, NFI > 0.90, IFI > 0.90, RFI > 0.90, AGFI > 0.80, and GFI > 0.90. Based on the results presented in Table 4, all fit indices for the nine-factor model demonstrated a significantly acceptable fit to the data.

### Internal consistency reliability

To evaluate the internal consistency reliability of the Persian version of the PDI-40, Cronbach’s alpha analysis was employed, yielding a value of 0.92. This indicates a high level of internal consistency, surpassing the value of 0.88 reported by Peters, Joseph et al. [51].

### Validity

### Test-retest reliability

Using a test-retest strategy in a sub-sample of 126 non-clinical participants over a two-week period, temporal stability was calculated at 0.84. This indicates a high level of consistency in the measurement over time (CI = 0.82–0.86).

### Convergent validity

The convergent validity of the PDI-40 was assessed by comparing it to the DASS-21 and CAPE-42. As shown in Table 5, the subscales of the PDI-40 demonstrated positive correlations with the subscales of the DASS-21, ranging from 0.012 to 0.38. Similarly, the subscales of the PDI-40 showed positive correlations with the subscales of the CAPE-42, ranging from 0.19 to 0.63. These findings indicate good convergent validity, as the PDI-40 aligns with and measures related constructs as measured by the DASS-21 and CAPE-42.

**Table 5:** Pearson correlation between PDI-40 with components of DASS-21, CAPE-42 in non-clinical participants (n = 764)

	F1	F2	F3	F4	F5	F6	F7	F8	F9	PDI Tot
Depression	0.038	0.309^**^	0.275^**^	0.251^**^	0.186^**^	0.285^**^	0.075	0.114^**^	0.012	0.246^**^
Stress	0.161^**^	0.389^**^	0.322^**^	0.212^**^	0.291^**^	0.300^**^	0.146^**^	0.206^**^	0.114^**^	0.345^**^
Anxiety	0.139^**^	0.291^**^	0.282^**^	0.224^**^	0.218^**^	0.265^**^	0.108^*^	0.131^**^	0.095^*^	0.281^**^
Positive	0.477^**^	0.516^**^	0.401^**^	0.367^**^	0.504^**^	0.554^**^	0.382^**^	0.431^**^	0.480^**^	0.632^**^
Negative	0.232^**^	0.436^**^	0.393^**^	0.400^**^	0.405^**^	0.395^**^	0.215^**^	0.277^**^	0.191^**^	0.456^**^
Depressive	0.253^**^	0.466^**^	0.390^**^	0.348^**^	0.406^**^	0.465^**^	0.213^**^	0.283^**^	0.205^**^	0.476^**^
CAPE-42 Total	0.380^**^	0.545^**^	0.455^**^	0.430^**^	0.509^**^	0.543^**^	0.320^**^	0.387^**^	0.350^**^	0.607^**^

Note: F1 = grandiosity; F2 = persecution; F3 = control; F4 = depersonalization; F5 = catastrophic ideation and thought broadcast; F6 = negative self; F7 = suspiciousness; F8 = thought disturbance; F9 = ideation of reference. *p < 0.05; **p < 0.01

### Comparing PDI-40 in Non-Clinical and Clinical Samples

A total of 126 psychotic patients participated in this stage of the study, and all of them completed the PDI questionnaire. The mean (SD) age of the psychotic participants was 24.02 (3.63). Differences between the non-clinical sample and psychotic sample in factor loadings on nine components were shown in Table 6.

**Table 6:** Item loading on nine components (non-clinical sample only), and significant differences between the non-clinical sample (n = 1276) and psychotic sample(n = 126)

PDI-40 Items	F1	F2	F3	F4	F5	F6	F7	F8	F9
ITEM 1***			0.686						
ITEM 2***			0.639						
ITEM 3								0.735	
ITEM 4***			0.667						
ITEM 5*			0.428						
ITEM 6***		0.623							
ITEM 7**									0.708
ITEM 8		0.644							
ITEM 9***		0.512							
ITEM 10***							0.438		
ITEM 11*		0.742							
ITEM 12		0.722							
ITEM 13		0.635							
ITEM 14***							0.760		
ITEM 15**		0.437							
ITEM 16***	0.723								
ITEM 17***	0.606								
ITEM 18***	0.572								
ITEM 19***	0.615								
ITEM 20***									0.495
ITEM 21	0.700								
ITEM 22**	0.636								
ITEM 23***	0.480								
ITEM 24***	0.462								
ITEM 25***	0.702								
ITEM 26***								0.438	
ITEM 27*					0.452				
ITEM 28					0.483				
ITEM 29						0.556			
ITEM 30**							0.422		
ITEM 31***						0.622			
ITEM 32						0.314			
ITEM 33				0.689					
ITEM 34**						0.659			
ITEM 35*				0.534					
ITEM 36***				0.652					
ITEM 37***					0.624				
ITEM 38***						0.449			
ITEM 39***					0.461				
ITEM 40***					0.524				

***Note***: PDI = Peters et al. Delusions Inventory; F1-9 = components of PDI; F1 = grandiosity; F2 = persecution; F3 = control; F4 = depersonalization; F5 = catastrophic ideation and thought broadcast; F6 = negative self; F7 = suspiciousness; F8 = thought disturbance; F9 = ideation of reference

Independent t-test between the psychotic and non-clinical samples on each item: Item (*) = P < 0.05*; P < 0.01**; P < 0.001***

**Table 7:** Comparisons between non-clinical (sample of study 1 n = 512 + study 2 n = 764) and psychotic (n = 126) samples on components of the nine PDI-40 factors

	Non-clinical (n = 1276)			Psychosis (n = 126)			
	Mean	SD	Median	Mean	SD	Median	value
PDI	117.75	90.20	102	178.63	97.48	164	50329.0***
F1	38.08	29.26	33	55.06	29.36	55	54230.0***
F2	30.89	24.58	26	38.93	21.60	38	61564.0***
F3	10.09	12.51	6	16.80	13.59	14	55654.0***
F4	6.54	9.26	0	9.85	11.29	8	68591.0***
F5	10.61	13.40	6	17.65	14.67	17	56573.0***
F6	9.82	13.18	5	18.30	15.78	16	52994.0***
F7	4.27	7.14	0	8.87	10.56	6	61765.0***
F8	4.36	6.12	0	6.22	6.49	6	66962.0***
F9	3.05	5.51	0	6.92	7.35	7	56934.0***

Note. PDI = Peters et al. Delusions Inventory; F1-9 = components of PDI; (*) = P < 0.05*; P < 0.01**; P < 0.001***(Mann-Whitney test between non-clinical and psychotic samples; 2 -tailed).

Table 7 presents significant differences between the non-clinical and psychotic samples. The psychotic samples obtained higher scores than the non-clinical participants on all components of the PDI-40. The evaluations of the diagnostic efficiency of the PDI-40 are presented below.

**Fig. 3:**
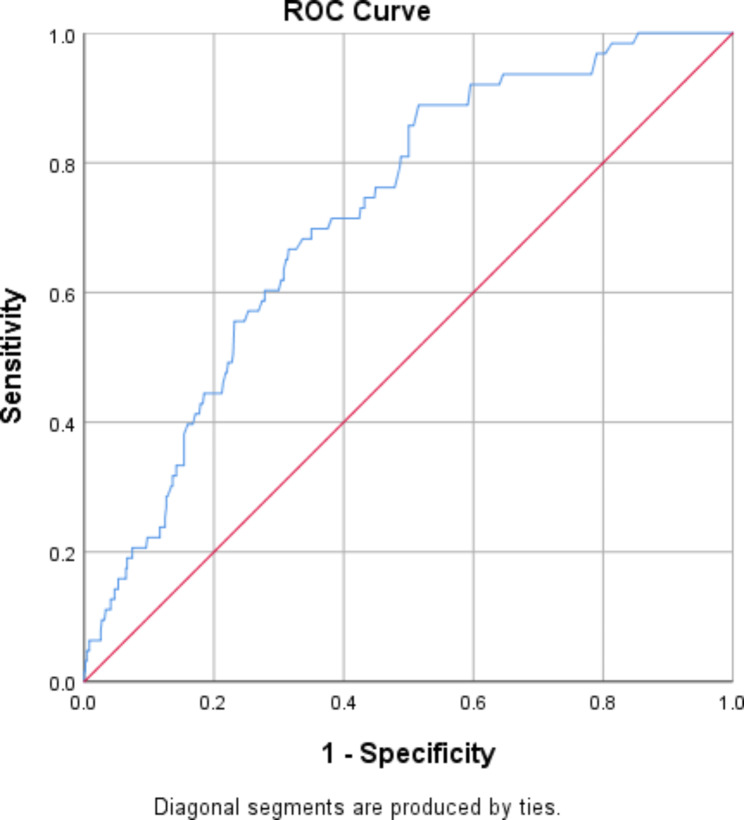
Receiver Operating Characteristic (ROC) curve of Peters et al. Delusions Inventory (PDI). The standard error (SE) was 0.03; the 95% confidence interval was 0.661– 0.780); Using a cutoff score of 107.80 or more, the sensitivity of the PDI was 0.73%, and the specificity was 0.4

Receiver Operating Characteristic (ROC) curves was utilized to examine the diagnostic performance of the Peters et al. Delusions Inventory (PDI-40) by analyzing clinical data. The area under the curve was 0.731 when using the PDI-40 to diagnose the psychotic group, indicating acceptable diagnostic capabilities. Similarly, the area under the curve was 0.426 when using the PDI-40 to diagnose the non-clinical sample. These results are presented in Fig. 3, highlighting the diagnostic potential of the PDI-40.

## Discussion

This study aimed to evaluate the reliability and validity of the Persian version of Peters et al. Delusions Inventory (PDI-40) in Iranian non-clinical and clinical samples. The findings provide confirmation that PDI-40 effectively assesses and categorizes delusion-proneness in non-clinical individuals. The scale demonstrated satisfactory internal coherence. To investigate the inherent factor structure of the PDI-40, a Principal Components Analysis (PCA) was executed, involving the inclusion of all items without any prior elimination. Notably, the analysis highlighted a noteworthy differentiation between the non-clinical and clinical samples in terms of the PDI-40 scores. This particular observation, while integral to our study’s objectives, diverges from the core focus of the PCA results. An evident and statistically significant distinction between the non-clinical and clinical samples was observed in relation to the PDI-40 scores. While this specific observation is not directly tied to the PCA itself, it does hold relevance within the broader context of our study’s objectives. The separation between non-clinical and clinical samples based on PDI-40 scores suggests potential clinical utility for this assessment tool in discerning between these cohorts. This outcome warrants further investigation and consideration in future research endeavors aimed at refining our understanding of the factors contributing to such differentiation.

In total, nine components were extracted from the PDI-40 using varimax rotation. It is worth noting that the intention behind the PDI-40 was not to measure a limited number of subscales, but rather to evaluate a broad range of delusions. Moreover, all items exhibited positive associations, indicating a shared underlying characteristic among them. The distribution of PDI-40 scores in our study exhibits a significant overlap. This overlap not only aligns with previous research conducted by Peters, Joseph et al. [65], Yeon Jung et al. [48] and Preti et al. [50], but also corroborates the findings of Kao et al. [49]. This alignment serves to fortify the robustness and consistency of our results, highlighting a convergence of evidence across diverse cultural and geographical contexts. To deepen our comprehension of the integration between our findings and the broader literature, we must delve into the contextual similarities and disparities that arise from this multifaceted perspective. By scrutinizing methodological congruence, conceptual parallels, and practical implications, we can illuminate the intricate relationship between our research and the existing body of work. Methodologically, our study is in alignment with the methodologies employed by Peters, Joseph et al., [65], Yeon Jung et al. [48], Preti et al. [50], and Kao et al. [49]. As Kao et al. [49], our research investigates the reliability, validity, and utility of the PDI within a specific cultural context. This shared methodological focus offers a comprehensive comprehension of the PDI’s performance in varying populations, enhancing its cross-cultural applicability. Our study resonates with the theoretical underpinnings of Peters, Joseph et al., [65] Yeon Jung et al. [48], Preti et al. [50], and Kao et al. [49] all of which contribute collectively to the evolving discourse on delusion proneness. By incorporating the PDI as a metric for psychosis proneness, we contribute to the nuanced understanding of this construct, while also extending its pertinence to diverse populations, as underscored by Kao et al. [49]. Regarding practical implications, the sensitivity and specificity values of our study, akin to Kao et al. [49], validate the utility of the PDI as a dependable diagnostic tool. Kao et al’s [49] establishment of specific yes/no cut-off scores for delusion proneness further buttresses the clinical applicability of the PDI, augmenting its potential to aid in identifying and distinguishing individuals with and without psychosis proneness. Our research underscores the alignment of our findings with prior investigations, including those by Peters, Joseph et al., [65], Yeon Jung et al. [48], Preti et al. [50], and Kao et al. [49]. These congruent values not only validate our results but also contribute to the broader discussions on the clinical applicability of the PDI-40.

Our study also found significant correlations between PDI-40 scores and the DASS-21 (Depression, Stress, and Anxiety) as well as the CAPE-42. Therefore, in accordance with prior studies, we can argue that higher levels of delusional beliefs are associated with increased levels of depression, anxiety, and stress [66–68] as well as a greater occurrence of PLEs [69–71]. The present research suggests that examining these dimensions can provide insight into an individual’s beliefs along the spectrum between mental health and psychosis.

The spectrum of delusional and psychotic experiences encompasses individuals from both the non-clinical population to clinical cases of psychosis at the opposite end. This perspective underscores the importance of comprehending the multidimensional nature of delusion proneness and its significance in terms of distress, preoccupation, and conviction, distinguishing between non-clinical individuals and those with psychosis [51]. Another crucial aspect to consider is the cultural context and societal acceptance of these beliefs within Iranian society. Unfortunately, the lack of relevant studies conducted in Iran makes it challenging to draw direct comparisons with the present findings. Additionally, in this study, no gender differences were observed regarding the frequency of beliefs and their dimensions, which aligns with findings from other studies [37, 51]. Finally, while the PDI-40 has demonstrated promising diagnostic capabilities, it is important to exercise caution when extrapolating its diagnostic potential solely from self-report measures. While the PDI-40 may indeed prove to be a valuable tool for mental health professionals, it should not be solely relied upon as the exclusive foundation for establishing a diagnosis. To strengthen the empirical basis of its diagnostic utility, additional evaluations using diverse samples representative of both non-clinical and clinical samples would be highly beneficial. This would provide a more comprehensive understanding of the scale’s effectiveness across a broader spectrum of individuals.

The validation of the Persian version of the PDI-40 in this study has significant implications for psychiatric research and clinical practice. The findings offer robust evidence supporting the utility and psychometric properties of the PDI-40 for assessing delusion proneness in both Iranian non-clinical and clinical samples. This study establishes the scale’s reliability, validity, and its capacity to differentiate between non-clinical and clinical groups, thereby paving the way for future investigations into delusion proneness and its potential associations with mental health and psychosis. Moreover, the factor analysis reveals the multidimensional nature of delusion proneness, with nine components identified. This underscores the complexity and heterogeneity of delusional experiences and emphasizes the importance of considering multiple dimensions, such as distress, preoccupation, and conviction, when assessing individuals along the mental health-psychosis continuum. Understanding these dimensions can provide valuable insights into an individual’s cognitive processes, beliefs, and subjective experiences, ultimately aiding in early detection, intervention, and treatment planning for individuals at risk of developing psychosis.

While this study represents a significant advancement within its field, it is imperative to acknowledge and address inherent limitations. Primarily, the clinical sample was exclusively drawn from a local hospital, which potentially restricts the generalizability of findings to a broader population of individuals grappling with psychosis. To enhance the robustness and external validity of future investigations, we recommend the inclusion of a more diverse and representative clinical cohort spanning various healthcare settings, encompassing both inpatient and outpatient populations. An important limitation arises from the reliance on self-report questionnaires, particularly the PDI-40, for assessing PLEs. This approach introduces potential constraints rooted in self-report bias and the subjective interpretation of questionnaire items. Although meticulous efforts were exerted to validate the Persian version of the PDI-40, establishing its reliability and convergent validity, future explorations should consider the integration of complementary assessment modalities. The incorporation of techniques such as clinician-administered interviews or observer-rated measures would supplement self-report data, enhancing the depth and comprehensiveness of our comprehension of delusion proneness. It is pertinent to recognize another inherent limitation of this study. The construct validity of the PDQ-40 could have been further elucidated through an exploration of gender and age invariance, factors that hold significance in comprehensively assessing the measure’s validity. The study refrained from employing desirability or infrequency of response scale to validate the results. While these aspects are acknowledged, they may potentially influence the validity of the findings, thus warranting meticulous consideration when interpreting the study’s outcomes.

This study contributes to the existing literature on delusion proneness by validating the Persian version of the PDI-40 and exploring its factor structure in an Iranian population. The use of a large sample size comprising both non-clinical and clinical samples strengthens the reliability and generalizability of the findings. The inclusion of convergent validity measures, such as the CAPE-42 and DASS-21, further supports the construct validity of the PDI-40. The identification of nine distinct components within the PDI-40 provides a comprehensive understanding of the various dimensions of delusion proneness.

The findings of this study also have significant clinical implications. The PDI-40 can serve as a valuable tool for clinicians in assessing and monitoring delusion proneness in individuals at different stages of mental health and psychosis. Early identification and intervention for individuals with high levels of delusion proneness may contribute to preventive strategies and reduce the risk of transitioning to clinical psychosis. The use of a validated and reliable tool like the PDI-40 enhances clinical decision-making and facilitates personalized treatment planning for individuals experiencing delusional beliefs.

An important avenue for future research lies in the exploration of cultural and societal influences on delusion proneness within the Iranian context. While our current study sheds light on the prevalence and psychological correlates of delusion proneness, there remains a research gap in understanding how cultural and societal factors contribute to the acceptance, interpretation, and management of delusional beliefs in Iran. Comparative investigations that delve into these intricate dynamics would provide valuable insights into the variations of delusion proneness across diverse societies, including Iran. By examining the interplay between cultural factors and delusion proneness, researchers can gain a deeper understanding of how cultural norms, beliefs, and social structures shape individuals’ experiences and expressions of delusions. This inquiry could encompass an analysis of how cultural narratives, historical contexts, and religious beliefs intersect with the manifestation and interpretation of delusional beliefs. Additionally, investigating the societal reception of individuals exhibiting delusions and their propensity to seek treatment can further illuminate the role of cultural and societal factors in influencing help-seeking behaviors and mental health outcomes.Our study contributes to this broader research agenda by highlighting the need for comprehensive investigations into the cultural dimensions of delusion proneness. by absence of comparative studies is indeed a limitation of the current work, it also underscores the potential for future studies to build upon our findings. By addressing this research gap, researchers can enhance the field’s understanding of the complex interplay between culture, society, and mental health, ultimately advancing both theoretical knowledge and practical interventions in the realm of delusion proneness.

Building upon the strengths of this study, future research should explore several avenues to advance our understanding of delusion proneness and its implications. Firstly, conducting longitudinal studies would be valuable in investigating the temporal stability and predictive validity of the PDI-40 for assessing the risk of developing psychosis. Longitudinal designs would allow researchers to track the trajectory of delusion proneness over time and explore potential associations with the onset of psychotic disorders. Additionally, given the cultural context of the Iranian population, further research should examine the cultural factors influencing the manifestation and interpretation of delusional beliefs. Comparative studies between different cultural groups would provide insights into the cultural specificity or universality of delusion proneness, contributing to the development of culturally sensitive assessment tools and intervention strategies.

Moreover, expanding the application of the PDI-40 to diverse populations, such as adolescents, the elderly, and outpatients, would enhance its generalizability and utility. Investigating the psychometric properties of the PDI-40 in these populations would provide valuable information for researchers and clinicians working with specific age groups or clinical settings. Furthermore, exploring the association between delusion proneness and other psychiatric conditions, such as personality disorders or substance use disorders, could shed light on the complex interplay between different mental health factors. Understanding these associations may have implications for differential diagnosis, treatment planning, and the development of targeted interventions for individuals with co-occurring disorders.

Lastly, considering the rapid advancements in technology, future studies could explore the potential of integrating digital assessments and wearable devices to monitor delusion proneness in real-time. The use of ecological momentary assessment methods and passive data collection could provide a more nuanced understanding of the contextual factors influencing delusion proneness and enable early intervention strategies tailored to individual needs.

In conclusion, our study highlights the potential of the Persian version of PDI-40 as a valuable tool for investigating both non-clinical individuals and clinical samples. By pursuing these outlined future directions, researchers can continue to deepen our understanding of delusion proneness, its underlying mechanisms, and its clinical implications. This comprehensive approach promises to pave the way for the development of effective prevention and intervention strategies, particularly for individuals at risk of psychosis, contributing to improved mental health outcomes across diverse populations.

## Data Availability

The corresponding author will provide the datasets generated and analyzed during this study upon a reasonable request.

## References

[1] Roehr B (2013). American Psychiatric Association explains DSM-5. BMJ.

[2] Cermolacce M, Sass L, Parnas J (2010). What is bizarre in bizarre delusions? A critical review. Schizophr Bull.

[3] Heilskov SER, Urfer-Parnas A, Nordgaard J (2020). Delusions in the general population: a systematic review with emphasis on methodology. Schizophr Res.

[4] Fusar-Poli L, Ciancio A, Gabbiadini A, Meo V, Patania F, Rodolico A (2020). Self-reported autistic traits using the AQ: a comparison between individuals with ASD, psychosis, and non-clinical controls. Brain Sci.

[5] Rognli EB, Bramness JG, Skurtveit S, Bukten A (2017). Substance use and sociodemographic background as risk factors for lifetime psychotic experiences in a non-clinical sample. J Subst Abuse Treat.

[6] Bell V, Halligan PW, Ellis HD (2008). Are anomalous perceptual experiences necessary for delusions?. J Nerv Ment Dis.

[7] Toutountzidis D, Gale TM, Irvine K, Sharma S, Laws KR (2022). Childhood trauma and schizotypy in non-clinical samples: a systematic review and meta-analysis. PLoS ONE.

[8] Castiajo P, Pinheiro AP (2021). Attention to voices is increased in non-clinical auditory verbal hallucinations irrespective of salience. Neuropsychologia.

[9] Freeman D, Startup H, Dunn G, Černis E, Wingham G, Pugh K (2013). The interaction of affective with psychotic processes: a test of the effects of worrying on working memory, jumping to conclusions, and anomalies of experience in patients with persecutory delusions. J Psychiatr Res.

[10] Shepherd AJ, Patterson AJK (2020). Exploration of anomalous perceptual experiences in migraine between attacks using the Cardiff anomalous perceptions scale. Conscious Cogn.

[11] Kelleher I, Cannon M (2011). Psychotic-like experiences in the general population: characterizing a high-risk group for psychosis. Psychol Med.

[12] Van J, Linscott RJ, Myin-Germeys I, Delespaul P, Krabbendam L (2009). A systematic review and meta-analysis of the psychosis continuum: evidence for a psychosis proneness–persistence–impairment model of psychotic disorder. Psychol Med.

[13] Rössler W, Riecher-Rössler A, Angst J, Murray R, Gamma A, Eich D (2007). Psychotic experiences in the general population: a twenty-year prospective community study. Schizophr Res.

[14] Loewy RL, Johnson JK, Cannon TD (2007). Self-report of attenuated psychotic experiences in a college population. Schizophr Res.

[15] Narita Z, Banawa R, Zhou S, DeVylder J, Koyanagi A, Oh H (2022). Loneliness and psychotic experiences among US university students: findings from the healthy minds study 2020. Psychiatry Res.

[16] Narita Z, Stickley A, DeVylder J (2020). Loneliness and psychotic experiences in a general population sample. Schizophr Res.

[17] Varghese D, Scott J, Welham J, Bor W, Najman J, O’Callaghan M (2011). Psychotic-like Experiences in Major Depression and anxiety Disorders: a Population-Based survey in young adults. Schizophr Bull.

[18] Hosseini SR, Tabbassi SS, Mosaferi S, Mousavinezad SH, Nooripour R, Firoozabadi A (2023). The Persian version of the psychotic-like Experiences Questionnaire for Children (PLEQ‐C): psychometric properties in iranian school students. Psychol Sch.

[19] Isaksson J, Vadlin S, Olofsdotter S, Åslund C, Nilsson KW (2020). Psychotic-like experiences during early adolescence predict symptoms of depression, anxiety, and conduct problems three years later: a community-based study. Schizophr Res.

[20] Gutteridge TP, Lang CP, Turner AM, Jacobs BW, Laurens KR (2020). Criterion validity of the psychotic-like Experiences Questionnaire for children (PLEQ-C). Schizophr Res.

[21] Hinterbuchinger B, Mossaheb N (2021). Psychotic-like Experiences: a challenge in Definition and Assessment. Front Psychiatry.

[22] Livet A, Navarri X, Potvin S, Conrod P (2020). Cognitive biases in individuals with psychotic-like experiences: a systematic review and a meta-analysis. Schizophr Res.

[23] Knežević G, Lazarević LB, Zorić A (2022). The meaning of momentary psychotic-like experiences in a non-clinical sample: a personality perspective. PLoS ONE.

[24] Lindgren M, Numminen L, Holm M, Therman S, Tuulio-Henriksson A (2022). Psychotic-like experiences of young adults in the general population predict mental disorders. Psychiatry Res.

[25] Espinosa A, Anglin DM, Pandit S (2022). Emotional self-efficacy informs the interrelation between discrimination, ethnic identity and psychotic-like experiences. Emotion.

[26] Tan M, Barkus E, Favelle S (2021). The cross-lagged relationship between loneliness, social support, and psychotic-like experiences in young adults. Cognit Neuropsychiatry.

[27] Fonseca-Pedrero E, Lemos-Giráldez S, Paino M, Muñiz J (2011). Schizotypy, emotional–behavioural problems and personality disorder traits in a non-clinical adolescent population. Psychiatry Res.

[28] Richardson T, Sood M, Bayliss P, Newman-Taylor K (2023). Self‐compassion as a mediator of the relationship between childhood sexual abuse and psychotic symptoms in clinical and non‐clinical groups. Br J Clin Psychol.

[29] Hinterbuchinger B, Koch M, Trimmel M, Litvan Z, Baumgartner J, Meyer EL (2023). Psychotic-like experiences in non-clinical subgroups with and without specific beliefs. BMC Psychiatry.

[30] McGrath JJ, Saha S, Al-Hamzawi A, Alonso J, Bromet EJ, Bruffaerts R (2015). Psychotic Experiences in the General Population: a cross-national analysis based on 31 261 respondents from 18 countries. JAMA Psychiatry.

[31] Linscott RJ, van Os J (2013). An updated and conservative systematic review and meta-analysis of epidemiological evidence on psychotic experiences in children and adults: on the pathway from proneness to persistence to dimensional expression across mental disorders. Psychol Med.

[32] Bourgin J, Tebeka S, Mallet J, Mazer N, Dubertret C, Le Strat Y (2020). Prevalence and correlates of psychotic-like experiences in the general population. Schizophr Res.

[33] Lu D, Qiu S, Xian D, Zhang J, Zhang Y, Liu X (2022). Psychotic-like experiences and associated socio-demographic factors among pregnant women in each trimester in China. Front Psychiatry.

[34] Stainton A, Chisholm K, Woodall T, Hallett D, Reniers RLEP, Lin A (2021). Gender differences in the experience of psychotic-like experiences and their associated factors: a study of adolescents from the general population. Schizophr Res.

[35] Schoorl J, Barbu MC, Shen X, Harris MR, Adams MJ, Whalley HC (2021). Grey and white matter associations of psychotic-like experiences in a general population sample (UK Biobank). Transl Psychiatry.

[36] Hafeez D, Yung AR (2021). Early persistence of psychotic-like experiences in a community sample of adolescents. Early Interv Psychiatry.

[37] Peters E, Joseph S, Garety PA (1999). Measurement of delusional ideation in the Normal Population: introducing the PDI (Peters et al. Delusions Inventory) Schizophr Bull.

[38] Peters ER, Joseph SA, Garety PA (1995). The measurement of delusional ideation in the normal population—-introducing the PDI (PEters et al. delusions inventory). Schizophr Res.

[39] Linney YM, Murray RM, Peters ER, MacDONALD AM, Rijsdijk F, Sham PC (2003). A quantitative genetic analysis of schizotypal personality traits. Psychol Med.

[40] Peters E, Day S, Mckenna J, Orbach G (1999). Delusional ideation in religious and psychotic populations. Br J Clin Psychol.

[41] Nunn JA, Rizza F, Peters ER (2001). The incidence of Schizotypy among Cannabis and Alcohol users. J Nerv Ment Dis.

[42] Schürhoff F, Szöke A, Méary A, Bellivier F, Rouillon F, Pauls D (2003). Familial aggregation of Delusional Proneness in Schizophrenia and bipolar pedigrees. Am J Psychiatry.

[43] Paíno-Piñeiro M, Fonseca-Pedrero E, Lemos-Giráldez S, Muñiz J (2008). Dimensionality of schizotypy in young people according to sex and age. Personal Individ Differ.

[44] Preti A, Marongiu S, Petretto DR, Miotto P, Masala C. Esperienze psichiche inusuali. Studio di validazione della versione italiana del Peters et al delusions inventory. Volume CXXVI. Riv Sper Freniatr; 2002.

[45] Prochwicz K, Gawęda Ł (2015). The polish version of the Peters et al. Delusions Inventory: factor analysis, reliability and the prevalence of delusion-like experiences in the polish population. Psychiatr Pol.

[46] López-Ilundain JM, Pérez-Nievas E, Otero M, Mata I (2006). Peter’s delusions inventory in spanish general population: internal reliability, factor structure and association with demographic variables (dimensionality of delusional ideation). Actas Esp Psiquiatr.

[47] Yamauchi T, Sudo A, Tanno Y (2007). Reliability and validity of the Japanese Version of Paranoia Checklist. Jpn J Personal.

[48] Yeon Jung H, Seung Chang J, Seo Yi J, Hwang S, Shin H-K, Hoon Kim J (2008). Measuring psychosis proneness in a nonclinical korean population: is the Peters et al delusions inventory useful for assessing high-risk individuals?. Compr Psychiatry.

[49] Kao Y-C, Wang T-S, Lu C-W, Cheng T-H, Liu Y-P et al. The psychometric properties of the Peters. Delusions Inventory (PDI) in Taiwan: reliability, validity, and utility. Soc Psychiatry Psychiatr Epidemiol. 2012;47:1221–34. 10.1007/s00127-011-0428-y 21861160

[50] Preti A, Rocchi MBL, Sisti D, Mura T, Manca S, Siddi S (2007). The psychometric discriminative properties of the Peters et al delusions inventory: a receiver operating characteristic curve analysis. Compr Psychiatry.

[51] Peters E, Joseph S, Day S, Garety P (2004). Measuring delusional ideation: the 21-Item Peters et al. Delusions inventory (PDI). Schizophr Bull.

[52] Ragazzi TCC, Shuhama R, Menezes PR, Del-Ben CM (2018). Cannabis use as a risk factor for psychotic-like experiences: a systematic review of non-clinical populations evaluated with the Community Assessment of psychic experiences. Early Interv Psychiatry.

[53] Stefanis NC, Hanssen M, Smirnis NK, Avramopoulos DA, Evdokimidis IK, Stefanis CN (2002). Evidence that three dimensions of psychosis have a distribution in the general population. Psychol Med.

[54] Rodgers B, Mann SA (1986). The reliability and validity of PSE assessments by lay interviewers: a national population survey. Psychol Med.

[55] Brenner K, Schmitz N, Pawliuk N, Fathalli F, Joober R, Ciampi A (2007). Validation of the English and french versions of the Community Assessment of psychic experiences (CAPE) with a Montreal community sample. Schizophr Res.

[56] Mirzaei Poueenak F, Ghanbari Pirkashani N, Nooripour R, Hosseini SR, Mazloomzadeh M, Shirkhani M. Psychometric validation of the Persian version of the community assessment of psychotic experiences-42 (CAPE-42) in iranian college students. Psychosis. 2021;:1–12.

[57] Lovibond PF, Lovibond SH (1995). The structure of negative emotional states: comparison of the Depression anxiety stress scales (DASS) with the Beck Depression and anxiety inventories. Behav Res Ther.

[58] Brown TA, Chorpita BF, Korotitsch W, Barlow DH (1997). Psychometric properties of the Depression anxiety stress scales (DASS) in clinical samples. Behav Res Ther.

[59] Yazdanshenas Ghazwin M, Kavian M, Ahmadloo M, Jarchi A, Golchin Javadi S, Latifi S (2016). The association between life satisfaction and the extent of Depression, anxiety and stress among iranian Nurses: a Multicenter Survey. Iran J Psychiatry.

[60] Shi D, Maydeu-Olivares A, Rosseel Y (2020). Assessing fit in Ordinal factor analysis models: SRMR vs. RMSEA. Struct Equ Model Multidiscip J.

[61] Kaiser HF (1970). A second generation little jiffy. Psychometrika.

[62] Bartlett MS (1954). A note on the multiplying factors for various χ ^2^ approximations. J R Stat Soc Ser B Methodol.

[63] Hau K-T, Marsh HW (2004). The use of item parcels in structural equation modelling: non-normal data and small sample sizes. Br J Math Stat Psychol.

[64] Tripodi SJ, Bender K (2011). Substance abuse treatment for juvenile offenders: a review of quasi-experimental and experimental research. J Crim Justice.

[65] Peters ER, Joseph SA, Garety PA (1999). Measurement of delusional ideation in the Normal Population: introducing the PDI (Peters et al. Delusions Inventory) Schizophr Bull.

[66] Freeman D, Emsley R, Dunn G, Fowler D, Bebbington P, Kuipers E (2015). The stress of the street for patients with persecutory delusions: a test of the symptomatic and psychological Effects of going outside into a busy urban area. Schizophr Bull.

[67] Meyer H, Taiminen T, Vuori T, Äijälä A, Helenius H (1999). Posttraumatic stress disorder symptoms related to psychosis and Acute Involuntary hospitalization in schizophrenic and delusional patients. J Nerv Ment Dis.

[68] Jones C, Griffiths RD, Humphris G, Skirrow PM (2001). Memory, delusions, and the development of acute posttraumatic stress disorder-related symptoms after intensive care. Crit Care Med.

[69] Schultze-Lutter F, Renner F, Paruch J, Julkowski D, Klosterkötter J, Ruhrmann S (2014). Self-reported psychotic-like experiences are a poor Estimate of Clinician-Rated attenuated and Frank Delusions and Hallucinations. Psychopathology.

[70] Varghese D, Scott J, McGrath J (2008). Correlates of Delusion-Like Experiences in a Non-Psychotic Community Sample. Aust N Z J Psychiatry.

[71] Bak M, Myin-Germeys I, Delespaul P, Vollebergh W, de Graaf R, van Os J (2005). Do different psychotic experiences differentially predict need for care in the general population?. Compr Psychiatry.

